# Application of microphysiological systems to unravel the mechanisms of schistosomiasis egg extravasation

**DOI:** 10.3389/fcimb.2025.1521265

**Published:** 2025-02-18

**Authors:** Martin Omondi Alfred, Lucy Ochola, Kennedy Okeyo, Euiwon Bae, Paul Ogongo, David Odongo, Kariuki Njaanake, J. Paul Robinson

**Affiliations:** ^1^ Department of Medical Microbiology and Immunology, University of Nairobi, Hospital Road, Kenyatta National Hospital, Nairobi, Kenya; ^2^ Department of Tropical and Infectious Diseases, Kenya Institute of Primate Research, Nairobi, Kenya; ^3^ Weldon School of Biomedical Engineering, Purdue University, West Lafayette, IN, United States; ^4^ Division of Experimental Medicine, Department of Medicine, University of California, San Francisco, San Francisco, CA, United States

**Keywords:** schistosomiasis, egg extravasation, granuloma, organ-on-a-chip (OOC), 3D microphysiological systems (3D MPS), animal models

## Abstract

Despite decades of control efforts, the prevalence of schistosomiasis remains high in many endemic regions, posing significant challenges to global health. One of the key factors contributing to the persistence of the disease is the complex life cycle of the *Schistosoma* parasite, the causative agent, which involves multiple stages of development and intricate interactions with its mammalian hosts and snails. Among the various stages of the parasite lifecycle, the deposition of eggs and their migration through host tissues is significant, as they initiate the onset of the disease pathology by inducing inflammatory reactions and tissue damage. However, our understanding of the mechanisms underlying *Schistosoma* egg extravasation remains limited, hindering efforts to develop effective interventions. Microphysiological systems, particularly organ-on-a-chip systems, offer a promising approach to study this phenomenon in a controlled experimental setting because they allow the replication of physiological microenvironments *in vitro*. This review provides an overview of schistosomiasis, introduces the concept of organ-on-a-chip technology, and discusses its potential applications in the field of schistosomiasis research.

## Introduction

Schistosomiasis, commonly referred to as bilharziasis, is a neglected tropical disease caused by parasitic trematodes of the genus *Schistosoma* which affects more than 251.4 million people worldwide, particularly in regions with limited resources including poor access to clean water (World Health Organization, 2023). The disease is endemic in tropical and subtropical regions, where it poses a significant burden on public health and socioeconomic development (Colley et al., 2014). Human schistosomiasis is caused by several species of *Schistosoma* parasites, the most predominant being *Schistosoma haematobium*, *Schistosoma mansoni*, and *Schistosoma japonicum*, each of which has a unique geographical distribution and clinical manifestation (Ross et al., 2002) along with other less prevalent species such as *Schistosoma mekongi*, *Schistosoma guineensis*, and *Schistosoma intercalatum*.

The life cycle of *Schistosoma* parasites involves two hosts: a definitive mammalian host and an intermediate host, usually a freshwater snail (McManus et al., 2018). Infection occurs when cercariae, the larval stage of the parasite, penetrates the human skin during contact with contaminated water. Once inside the human host, the cercariae metamorphose into a schistosomulum, and develop into adult worms that pair up as male and female couples that reside in the blood vessels surrounding the intestines (*S. mansoni* and *S. japonicum*) or bladder (*S. haematobium*). Female worms produce hundreds of eggs per day, which are then released into the bloodstream or lymphatic system (Gryseels et al., 2006).

Central to the pathogenesis of schistosomiasis is the process of egg deposition and extravasation within the host’s tissues. The eggs must cross the endothelial barrier of blood vessels to reach the lumen of the intestine or bladder, where they are excreted in stool or urine, respectively (Chitsulo et al., 2000). However, some eggs become trapped in tissues, leading to the formation of granulomas and fibrosis, which are hallmarks of chronic schistosomiasis. These pathological changes can result in severe morbidity, including liver and spleen enlargement, bladder cancer, and neurological complications (Chitsulo et al., 2000).

Despite decades of research, the molecular and cellular mechanisms underlying *Schistosoma* egg extravasation are incompletely understood. In part, this can be attributed to overreliance on animal models, which, due to their nature of complexity, do not allow the high-resolution studies necessary to decipher molecular mechanisms. Although animal experiments are indispensable, especially for preclinical screening in the drug discovery process, various issues, such as ethical considerations and species differences, remain (Kimura et al., 2018). To circumvent these challenges, cell-based assays using human-derived cells have been actively pursued. Furthermore, in recent years, there has been a growing interest in leveraging advanced biomedical technologies to overcome these limitations and unravel the “mysteries” of schistosome egg extravasation. To this end, organ-on-a-chip (OOC) platforms have emerged as powerful tools for recapitulating the physiological microenvironments of human organs *in vitro* (Bhatia and Ingber, 2014). OOC models offer unique advantages by providing dynamic multicellular systems that mimic the structural and functional complexity of human tissues. These *in vitro* models can provide valuable information on the pathogenesis of schistosomiasis, as they offer the simplicity of high-resolution imaging, allowing studying the molecular mechanisms of the disease in detail (Yeh et al., 2022; Yeh et al., 2024). Furthermore, OOCs are devoid of ethical and practical considerations that often restrict the use of human participants and animal models in the research of schistosomiasis.

This review explores the utility of OOC systems in unraveling the process of extravasation of *S. mansoni* eggs in humans. By systematically analyzing the existing literature, we identify key findings, knowledge gaps, and future research directions in this rapidly evolving field. Through a comprehensive examination of OOC-based studies, our objective is to elucidate the potential of this innovative technology to advance our understanding of the pathogenesis of schistosomiasis focusing on the role of immune cells in granuloma formation that could facilitate the development of novel therapeutic strategies.

## Schistosomiasis life cycle

**Figure 1:**
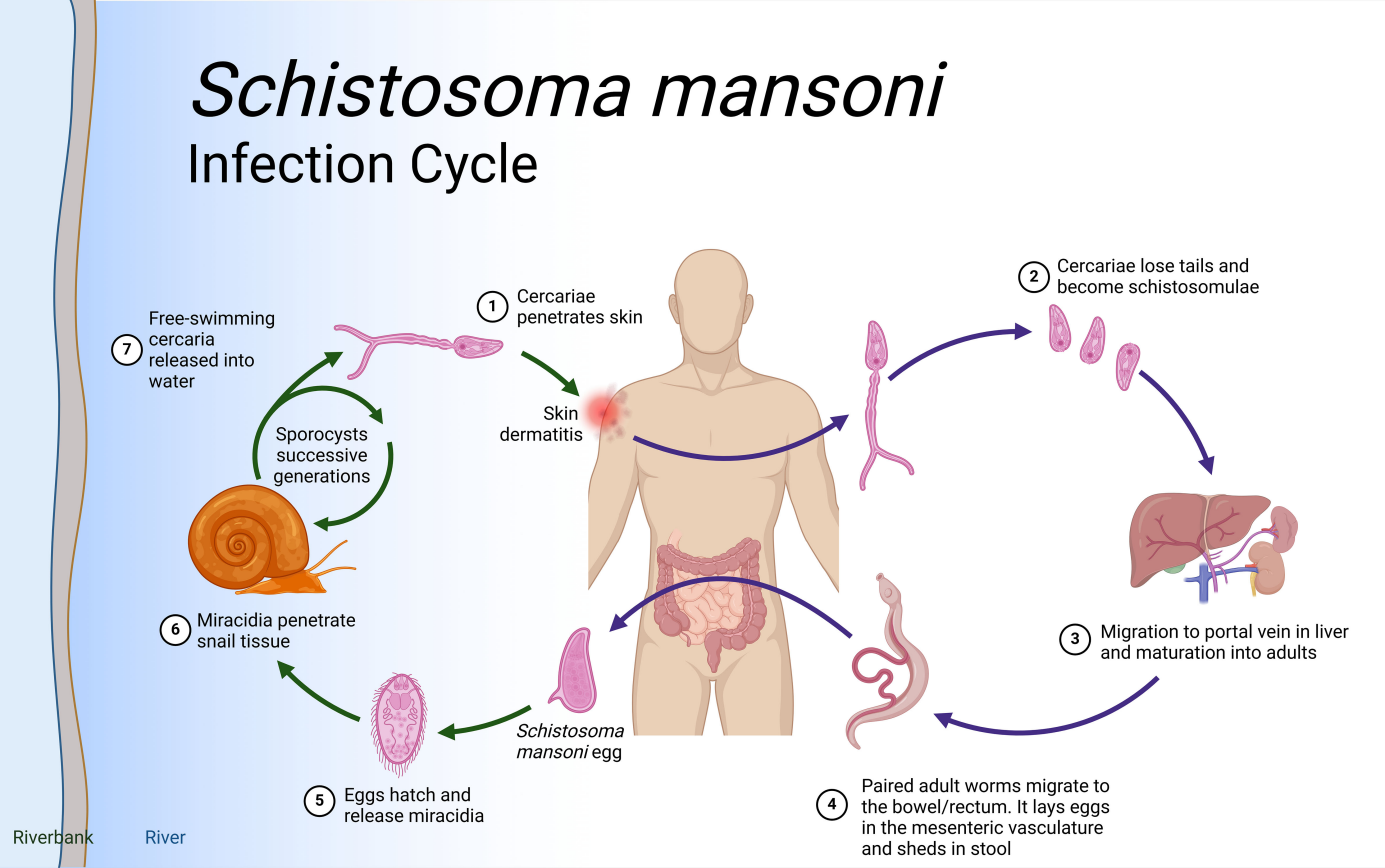
*Schistosoma* life cycle. Infection begins when (Step 1) free-living cercariae penetrate human skin and (Step 2) cercariae lose tails and become schistosomula, followed by (Step 3) migration through the portal vein in the liver and maturation to adult schistosomes. Once in the liver, the female and male adults pair up (Step 4) and migrate to the intestines where the females release eggs. Interaction with the microbiome, epithelial cell death and remodeling lead to the active release of eggs, which are then released to the environment with host feces. (Step 5). Lastly, when in water eggs hatch into miracidia (step 6) which eventually penetrate the tissue of Biomphalaria spp snails to continue the cycle (step 7). Eggs that do not extravasate are encapsulated by vascular endothelia cells that trigger intravascular host-immune responses to induce VEC inflammation, proliferation, and migration. This then leads to the formation of granuloma in the liver that results in liver fibrosis after about 8 weeks. Created in BioRender.com.

*Schistosoma* species have intricate life cycles involving both a freshwater snail as an intermediate host and a mammalian host, such as humans, as the definitive host as illustrated in **Figure 1**. *S. mansoni* eggs are expelled through feces, while *S. haematobium* eggs exit via urine. Upon reaching water body, these eggs hatch under favorable conditions such as temperature, pH and turbidity. In snails (e.g., *Biomphalaria* for *S. mansoni*), asexual reproduction produces large numbers of free-swimming cercariae, which actively seek out and penetrate the skin of a mammalian host, developing into schistosomula. Initially residing in the skin, schistosomula then migrate to the bloodstream and, within 5-7 days, reach the lungs. After circulating for about two weeks, they settle in the hepatoportal system for *S. mansoni* and veins of the bladder and the pelvic organs for *S. haematobium*, where they sexually mature.

Mature worm pairs migrate to the mesenteric veins, with adult *S. mansoni* worms primarily residing in the inferior mesenteric veins surrounding the colon and caecum. Female worms lay eggs in capillary walls, where they either enter the bloodstream or move through the intestinal epithelium into the lumen. These metabolically active and immunogenic eggs provoke an inflammatory response, resulting in granuloma formation that aids their passage through intestinal tissues. The life cycle is completed when eggs are excreted in feces. In the case of *S. haematobium*, once matured, the female worm lays eggs in the blood vessels. The eggs move through the bladder wall and are expelled with urine, continuing the cycle. Some eggs, however, become trapped in surrounding tissues, causing inflammation and leading to urogenital pathology such as bladder fibrosis, calcification, and, over time, an increased risk of bladder cancer.

Acute schistosomiasis may present as a mild skin rash (swimmer’s itch) and systemic symptoms like fever, fatigue, and cough (Katayama fever). The pathological hallmark of schistosomiasis is the immunopathology associated with egg deposition in host tissues and their subsequent excretion. Vascular endothelial cells (VECs) encapsulate these eggs, triggering an inflammatory granulomatous response that facilitates egg extravasation (Yeh et al., 2022; Yeh et al., 2024). Some eggs fail to reach the intestines or bladder, becoming trapped in host tissues and forming granulomas, which consist of immune cells, fibroblasts, and collagen. These granulomas are characteristic of chronic schistosomiasis, contributing to tissue damage, fibrosis, and organ dysfunction (Pearce and MacDonald, 2002). This can cause portal hypertension, gastrointestinal bleeding, hepatic encephalopathy, and liver failure.

## Gaps in our understanding of extravasation of *S. mansoni* eggs

**Figure 2:**
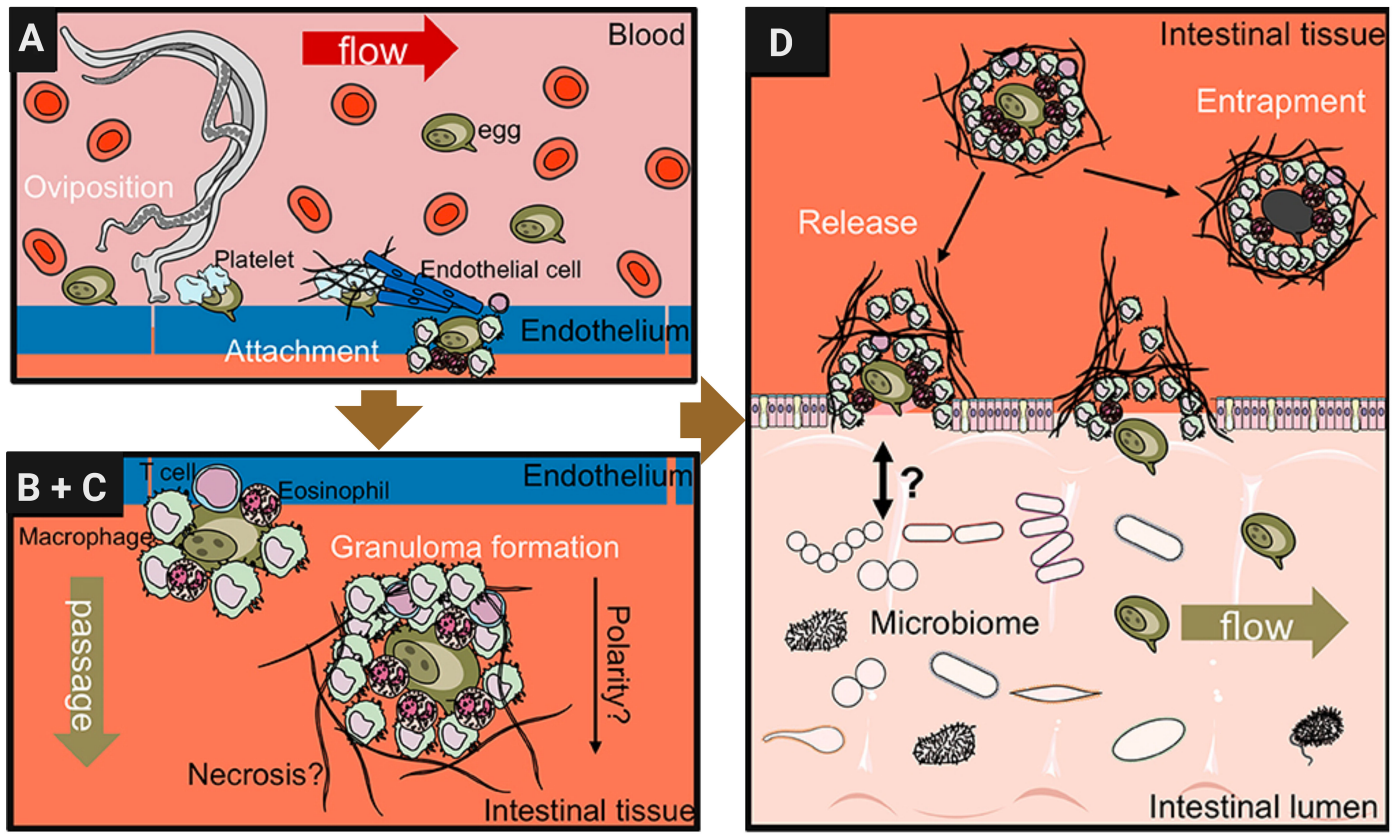
Schistosome egg transition through gastrointestinal tissues. Modified from Schwartz and Fallon (2018) with further editing in Biorender.com. **(A)** Adult female schistosomes deposit eggs (about 300 eggs per day for *S. mansoni*) into the vasculature close to the lamina propria. Platelets and fibrinogen adhere to the eggs and activate the endothelium. Endothelial cells actively grow over the egg supporting its extravasation. Eggs that do not cross the endothelial border are disseminated by the blood flow and become trapped mostly in the liver portal system. **(B, C)** Immune cells, such as macrophages, T cells and eosinophils start to encapsulate the egg. Granuloma formation occurs around the egg and together with other processes, such as fibrinolysis, egg secretions-induced necrosis, leading to the passage of the egg toward the intestinal lumen. **(D)** Entrapped eggs become fibrotic and calcified in the liver during chronic schistosomiasis infection.

The *S. mansoni* egg proteome has been characterized in many studies but the pathogenic consequences of the interaction of egg molecules with the host immune system, as well as the processes that underlie the passage of eggs through tissues and their subsequent release with the feces, are still poorly understood (Schwartz and Fallon, 2018). Mechanisms by which eggs travel through tissues of the intestinal wall are believed to include modulation of local immune responses that favor their migration to the intestinal lumen. This mechanism by which schistosome eggs extravasate from the bloodstream into host tissues is complex and involves multiple steps as proposed in **Figure 2** (Schwartz and Fallon, 2018). When reaching the small blood vessels of the liver or other organs, the eggs lodge in the endothelium (Wynn and Cheever, 1995). This triggers a cascade of inflammatory responses characterized by the recruitment of immune cells and the release of cytokines and chemokines (Chiaramonte et al., 1999). Macrophages, neutrophils, eosinophils, and other immune cells are recruited to the site of deposition, where they interact with the endothelium and surrounding tissues (Colley and Secor, 2014). The production of pro-inflammatory cytokines, such as interleukin-1β (IL-1β), tumor necrosis factor-alpha (TNF-α), and interleukin-6 (IL-6), promotes endothelial activation and permeability, facilitating egg extravasation (Mpotje, 2017).

In addition, basophils can be activated directly by Immunomodulatory protein secreted by schistosomes alpha 1 (IPSE/α1) to release Interleukin (IL-4) that is present in intestinal granulomas. Recent studies have uncovered a novel molecular mechanism in which IPSE, a member of the βγ-crystallin superfamily, binds to Immunoglobulin E (IgE) via its crystallin fold. This interaction activates basophils independently of traditional IgE cross-linking (Cass et al., 2007). Once outside the bloodstream, eggs interact with host cells and components of the extracellular matrix (ECM), triggering additional inflammatory and fibrotic responses (Costain et al., 2018). Immune cells, particularly macrophages and eosinophils, recognize and phagocytose eggs, releasing cytokines and chemokines that recruit additional immune cells to the site of deposition (Pearce and MacDonald, 2002). Fibroblasts are activated to produce collagen and other ECM proteins, to complete the formation of granulomas (Colley and Secor, 2014). The size and composition of the granulomas vary depending on the number and location of the trapped eggs and the immune response of the host (Cass et al., 2007).

## *In vitro* immunological studies in schistosomiasis

One of the most intriguing aspects of schistosomiasis is its ability to evade the host immune system. Various studies have used *in vitro* models to investigate how *Schistosoma* spp. manipulates host immune responses. In a notable study, Kazura et al., 1981 used an *in vitro* approach to demonstrate that *S. mansoni* schistosomula releases excretory-secretory (ES) products that inhibit the production of reactive oxygen species (ROS) by human neutrophils. ROS are typically a key part of the immune response to pathogens, and the ability of the parasite to suppress ROS production aids in evasion of immune defenses. This study laid the foundation for further research into the role of ES products in immune evasion (Kazura et al., 1981). More recently, *in vitro* assays have shown that the extracellular vesicles (EVs) secreted by schistosomula play an essential role in immune modulation. These studies have demonstrated that EVs carry microRNAs (miRNAs) capable of altering host gene expression and effectively dampening pro-inflammatory responses. Their findings suggested that schistosomula-derived EVs can downregulate the expression of genes involved in innate immune signaling, helping the parasite establish an infection (Wu et al., 2019; Kuipers et al., 2020; Keshtkar et al., 2022).

Schistosome eggs are highly immunogenic and play a critical role in shaping immune responses during infection. An earlier study by Linder (2017) established that perioval granuloma formation is driven by the host immune responses where immune cells form protective structures around the eggs and this shields tissues from damage. Also, this process leads to significant fibrosis and pathology. The immune system responds to egg antigens by activating cells like macrophages and eosinophils, which initiate inflammation and help push the eggs through tissue barriers. The study also stated that mechanical forces associated with muscle contraction aid in egg movement to the perivascular space. Coagulation and fibrinolysis are also essential, as balanced clot formation and breakdown facilitate egg movement without excessive clotting or bleeding. The study distinguished between “successful” eggs, which reach the gut or bladder for excretion, and “unsuccessful” eggs, which become trapped in tissues, causing chronic inflammation and fibrosis (Linder, 2017).

Many studies have focused on understanding the immunomodulatory properties of egg-secreted proteins. For example, Pearce et al., 2005 identified specific egg antigens, including omega-1 (ω1), which are responsible for driving the T helper type 2 (Th2) immune response, a hallmark of chronic schistosomiasis. Using *in vitro* assays with dendritic cells, they showed that ω1, a glycoprotein secreted by schistosome eggs, induces the production of interleukin-4 (IL-4) by T helper cells. This discovery highlights the central role of egg antigens in skewing the immune response toward a Th2 phenotype, which is associated with granuloma formation and tissue fibrosis (Pearce, 2005). In this study, it was also found that egg antigens induce dendritic cells to produce thymic stromal lymphopoietin (TSLP), a cytokine that primes dendritic cells to support the development of Th2 responses. This provides additional mechanistic insight into how schistosome eggs drive chronic immune activation (Pearce, 2005).

The immune response to schistosome infection is tightly regulated to balance effective defense mechanisms and prevent excessive tissue damage. Regulatory T cells (Tregs) play a pivotal role in modulating immune responses during schistosome infections. Layland et al., 2007 conducted *in vitro* and *ex vivo* experiments to investigate the role of Tregs in schistosomiasis. They showed that during chronic infection, Tregs expand in response to schistosome egg antigens and are essential for controlling the size of granulomas. *In vitro* assays demonstrated that granuloma formation became more pronounced when Tregs were depleted, leading to increased tissue damage. This study highlights the importance of Tregs in preventing excessive immunopathology during schistosomiasis (Layland et al., 2007). More recently, studies have shown use of *in vitro* co-culture systems to investigate the effects of schistosome-derived products on the differentiation and function of Tregs. These findings revealed that schistosome eggs secrete molecules that directly promote the expansion of Tregs, thereby modulating the host immune environment to favor parasite survival, while limiting inflammation and pathology (Mu et al., 2021).

Granuloma formation around the schistosome eggs is a key pathological feature of chronic schistosomiasis. *In vitro* models have been developed to study cellular and molecular processes underlying granuloma formation. In an important study Zouain et al., 2004 used an *in vitro* granuloma model to investigate how *S mansoni* PIII antigen influences granuloma size and composition. They found that granulomas formed around schistosome eggs *in vitro* are dependent on the presence of IL-13, a cytokine that promotes fibrosis. This study provides direct evidence linking IL-13 levels to the fibrotic pathology observed in schistosomiasis (Zouain et al., 2004). Furthermore, studies have shown that schistosome egg antigens drive macrophages toward an alternatively activated (M2) phenotype, which is associated with tissue repair and fibrosis *in vitro*. These findings help explain the dual role of macrophages in controlling infection and contributing to pathology (Pearce and MacDonald, 2002).

In addition to immune modulation, schistosomes release proteolytic enzymes, which facilitate tissue invasion and egg translocation. Several *in vitro* assays have been used to explore the role of these proteases in schistosome biology. Felleisen et al., 1990 conducted *in vitro* assays using recombinant schistosome proteases to study their role in degrading host extracellular matrix proteins. They showed that the protease cathepsin B, secreted by schistosomula, is crucial for the ability of the parasite to enter the circulatory system (blood vessels or lymphatics) and are carried passively by the bloodstream to establish infection. This study highlights the importance of proteolytic enzymes in the early stages of infection (Felleisen and Klinkert, 1990).

## Animal models in schistosomiasis research

Human studies on schistosomiasis are typically conducted in endemic regions, and often focus on observations before and after treatment. While analyzing human samples, such as blood, tissue, and stool, is essential for translating animal research into human systems (Colley and Secor, 2014), these samples have limitations, including variability in genetic background, medical history, co-infections, and environmental factors. Moreover, ongoing studies using controlled human infection with *S. mansoni* promise to shed new light on many aspects of human infection (Janse et al., 2018). However, ethically studying the egg excretion process in humans poses challenges, particularly when considering deliberate chronic infections involving male and female worms, which can lead to egg production, tissue damage, and significant health risks. Longitudinal studies in endemic areas could be an alternative, although they present logistical difficulties, such as the need for colonoscopy, to examine the intestinal epithelium.

Animal models have played a critical role in the investigation of schistosomiasis, facilitating the study of parasite biology, host immune responses, disease pathogenesis, drug efficacy, and vaccine development. Various animal species have been used to model distinct stages of *Schistosoma* infection, each offering unique advantages in understanding the disease, as shown in **Table 1**. Baboons and chimpanzees are the most accurate models, replicating key features of human schistosomiasis, such as peri-portal fibrosis and intestinal lesions (Sadun et al., 1966; Abe et al., 1993; Yole, 1996; Nyindo and Farah, 1999; Farah et al., 2000) but these large animal models are costly with stricter ethical considerations. Despite some differences, particularly in hepatic fibrosis and pathology, which are more closely linked to the granulomatous response to trapped parasite eggs in the liver and intestine in mice (Fallon, 2000), the formation of granulomas in mice is still a valuable reflection of human disease. However, despite decades of research, the immunological and molecular mechanisms underlying the extravasation of schistosome eggs remain poorly understood.

**Table 1:** Selected schistosomiasis studies in animal models.

Research Area	Animal Model	Purpose/Findings	References
Parasite Biology and Lifecycle Studies	Mice, Hamsters, Nonhuman primates	The migration of *Schistosoma* from the skin to the liver and organs during infection was studied, as well as the cycles of egg laying.	(Lambertucci et al., 2014; Pereira et al., 2015; Wang et al., 2018; Lombardo et al., 2019; Maciel et al., 2019)
	Snail (*Biomphalaria* spp.)	Investigated the stages of life of *S. mansoni* in snails, including cercarial shedding and miracidial infection, establishment of infection, and factors such as temperature and genetics.	(Wang et al., 2016; Mutuku et al., 2017; Queiroz et al., 2017; Hambrook et al., 2018; Nguyen et al., 2021; Phan et al., 2022)
	Rodents (rats)	Examined the early development of schistosomula in rodent models to better understand parasite-host interactions.	(Han et al., 2013; Krautz-Peterson et al., 2017; Phan et al., 2022)
Pathogenesis of Schistosomiasis	Mice and Hamsters	Investigated the formation, fibrosis, and deposition of eggs in the liver and intestines of infected animals. Hepatosplenomegaly and portal vein obstruction due to chronic schistosomiasis infection.	(Warren, 1966; el-Sebai et al., 1989; Moustafa et al., 1996; Tiggelman et al., 1996; Abou Rashed et al., 1997; Wynn et al., 2004; Abouel-Nour et al., 2005; Helmy et al., 2005; Bartley et al., 2006; Oloris et al., 2007; Andrade and Santana, 2010; Sanches et al., 2020; Tang et al., 2020; Salama et al., 2021)
	Non-human primates (baboons)	Investigated liver fibrosis progression and schistosomiasis-associated cirrhosis.	(Andrade, 2004; Kariuki et al., 2004)
Host Immune Responses	Mice (BALB/c, C57BL/6, RAG-1 knockout), rats	Dissected the roles of T cells, macrophages, and eosinophils in the immune response and formation of granulomas, immune response during co-infection including vaccine response.	(Rumbley et al., 1998; Chiu and Chensue, 2002; MacDonald and Pearce, 2002; Burke et al., 2010; Chen et al., 2012; Joyce et al., 2012; Teixeira de Melo et al., 2013; Yu et al., 2015; Zhang et al., 2015; Zhu et al., 2017; Huang H. et al., 2022; Shen et al., 2022; Reinholdt et al., 2023)
	Guinea pigs	The authors studied delayed hypersensitivity responses to Schistosoma antigens, providing information on immune mechanisms.	(Dean et al., 1975; Pearce and McLaren, 1983; Gordon and McLaren, 1988)
	Non-human primates (baboons, rhesus monkeys)	Chronic infection model to assess immune modulation and cytokine response.	(Kariuki et al., 2004; Carvalho-Queiroz et al., 2015; Pearson et al., 2015; Nyakundi et al., 2016; Gent et al., 2019; Nyakundi et al., 2022)
Vaccine development	Mice (various strains)	Various vaccine candidates were tested, such as paramyosin, glutathione-S-transferase (GST) and the Sm-p80 antigen.	(El Ridi and Tallima, 2009; Frantz et al., 2011; Sulbarán et al., 2013; Molehin et al., 2021; Mossallam et al., 2021; de Oliveira Santos Bernardes et al., 2022; Lam et al., 2022; Tedla et al., 2022; Hassan et al., 2023; Elguindy et al., 2024)
	Baboons, Rhesus monkeys	Showed reduced worm burdens and egg production in vaccinated primates with recombinant antigens.	(Kariuki et al., 2004; Kariuki et al., 2004; Ahmad et al., 2011; Carvalho-Queiroz et al., 2015; Zhang et al., 2018; Gent et al., 2019)
Drug Testing and Efficacy Studies	Mice (various strains)	Praziquantel, oxamniquine, and new derivatives tested for efficacy and resistance development.	(Botros et al., 2011; Muchirah, 2012; Allan et al., 2014; Abla et al., 2017; Pinto et al., 2019; Nono et al., 2020)
	Hamsters	Novel antischistosomal compounds and their impact on parasite life stages and host immune response.	(Abdulla et al., 2009; Padalino et al., 2021; Alghamdi et al., 2023)
	Baboons	Assessed pharmacokinetics and drug resistance patterns, showing effective reduction of worms with praziquantel.	(Pica-Mattoccia and Cioli, 2004; Greigert et al., 2019; Melkus et al., 2020)
Schistosomiasis-Associated Comorbidities	Mice, Hamsters, and Rats	Investigated the role of chronic inflammation in the development of colorectal cancer in *Schistosoma*-infected mice. Effect of *Schistosoma* infection on the gut microbiome. Studied portal hypertension and esophageal varices associated with advanced schistosomiasis infection.	(Sarin et al., 1991; Tanaka, 2012; Hu et al., 2020)
	Non-human primates (baboons)	Modeled pulmonary hypertension and cardiovascular complications caused by schistosomiasis.	(Farah et al., 2013)
Schistosomiasis Transmission Dynamics	Snails (*Biomphalaria* spp.)	Investigated the role of environmental factors and snail control measures in interrupting transmission cycles.Modeled transmission in endemic regions to study the impact of water management and human-snail-parasite interactions.	(Sengupta et al., 2019; Huang Q. et al., 2022)

## OOC systems for modeling human physiology

OOC systems as summarized in **Table 2** represents a revolutionary approach to modeling human physiology *in vitro* by recreating the structural and functional complexity of human organs and tissues (Huh et al., 2013). These microfluidic devices, also known as 3D microphysiological systems (3D MPS), offer a unique platform for studying biological processes in a controlled environment that closely mimics the *in vivo* microenvironment (Bhatia and Ingber, 2014). OOC systems revolves around the creation of microscale devices that replicate the essential features of specific organs or tissues (Esch et al., 2014). These systems typically consist of microfluidic channels lined with living cells that simulate the structure and function of the target tissue (Bein et al., 2018). By culturing cells in a 3D microenvironment, OOC models provide more physiologically relevant conditions than traditional two-dimensional (2D) cell culture systems (Jiang et al., 2019).

**Table 2:** Current organ on a chip/disease models.

Organ/Model	Diseases studied	Key Features	Significance	References
Liver-on-a-Chip	Drug-induced liver injury, Hepatitis B, genotoxicity	Replicates liver metabolism, bile secretion, and cellular microarchitecture. Can be perfused with blood-like fluids for long-term studies.	Crucial for evaluating the hepatotoxicity of pharmaceuticals and studying liver diseases such as viral hepatitis. Can predict drug-induced liver injury (DILI).	(Shah et al., 2018; Beaurivage et al., 2019; Etxeberria et al., 2022; Kanabekova et al., 2022; Yang et al., 2023; Ewing et al., 2024)
Heart-on-a-Chip	Cardiotoxicity, Heart disease	Mimic heart tissue contraction, beat rate, and electrophysiology using human-induced pluripotent stem cells (hiPSC).	It is useful for studying cardiomyopathies and screening drug effects on heart function, for example, arrhythmias induced by certain cancer drugs.	(Morimoto et al., 2016; Zhang et al., 2021; Schneider et al., 2022)
Lung-on-a-Chip	Pulmonary infections, COPD, asthma	Simulates the alveolar-capillary interface with mechanical stretching to mimic breathing. Can expose epithelial cells to pathogens or allergens.	Enables the study of lung inflammatory diseases such as COPD, asthma, and infectious diseases such as tuberculosis.	(Huh, 2015; Müller et al., 2021; Shah et al., 2024)
Kidney-on-a-Chip	Kidney disease, Nephrotoxicity	Models renal tubular reabsorption and secretion, including filtration and electrolyte balance.	Provides information on nephrotoxicity caused by medications (eg, chemotherapeutics) and models diseases such as acute kidney injury (AKI).	(Guo et al., 2020; Sun et al., 2020; Chambers et al., 2023)
Gut-on-a-chip	Inflammatory Bowel Disease (IBD), Celiac Disease	Mimics intestinal peristalsis, villi structure, and microbe-host interactions. Incorporates epithelial, immune, and microbial cells.	Useful for studying gastrointestinal disorders such as Crohn’s disease, ulcerative colitis, and the role of the gut microbiome in health and disease.	(Beaurivage et al., 2019; Xian et al., 2023; Yang et al., 2023; Taavitsainen et al., 2024)
Brain-on-a-Chip	Alzheimer’s Disease, Parkinson’s Disease	Recreates blood-brain barrier (BBB) function and neuron-astrocyte interactions. Allows the study of neurotransmitter release and synaptic function.	A valuable model for studying neurodegenerative diseases such as Alzheimer’s and Parkinson’s, and for screening for neuroprotective drugs.	(van der Helm et al., 2016; Lenoir et al., 2022; de Rus Jacquet et al., 2023; Hall and Bendtsen, 2023)
Pancreas-on-a-Chip	Diabetes, Islet cell function	Mimics pancreatic islets and insulin secretion dynamics. Integrates endocrine tissue from human islet cells.	Enables the study of diabetes and glucose regulation, with potential applications in drug development for insulin production and beta-cell regeneration.	(Essaouiba et al., 2020a; Essaouiba et al., 2020b; Zandi Shafagh et al., 2022; Fernández-Costa et al., 2023; Kim et al., 2023)
Skin-on-a-Chip	Wound Healing, Psoriasis	Models of epidermal and dermal layers, with barrier function and nutrient delivery. Can include immune cells for inflammation studies.	Useful for studying skin diseases, including psoriasis, eczema, and for testing wound healing and drug delivery systems for topical applications.	(Wufuer et al., 2016; Quan et al., 2022; Sun et al., 2022; Cho et al., 2024)
Bone Marrow-on-a-Chip	Leukemia, Hematopoiesis	Models the bone marrow niche, hematopoietic stem cell (HSC) differentiation, and blood cell production under various conditions.	Helps in the study of blood cancers (e.g., leukemia) and hematopoiesis, allowing drug testing and information on the immune system response.	(Torisawa et al., 2014; Kefallinou et al., 2020; Ma et al., 2020; Nasello et al., 2021; Nelson et al., 2021)
Placenta-on-a-chip	Maternal-Fetal Interface, Preeclampsia	Simulates nutrient, oxygen, and drug transport between maternal and fetal cells. Recreates trophoblast-endothelial interactions.	Helps to understand placental diseases, drug effects during pregnancy, and conditions such as preeclampsia. Provides a safer model for drug testing.	(Blundell et al., 2016; Blundell et al., 2018; Yin et al., 2019; Rahman et al., 2024)

The key advantage of OOC system is its ability to simulate dynamic microenvironments, including fluid flow, mechanical forces, and cell-cell interactions (Huh et al., 2010). The microfluidic channels embedded within the device allow precise control over the delivery of nutrients, oxygen, and signaling molecules to cultured cells, mimicking the physiological conditions found *in vivo* (Esch et al., 2015). OOC systems are suitable for studying complex biological processes such as host-pathogen interactions. Unlike animal models, OOC systems allow the use of human cells, reducing the need for animal experimentation and improving the relevance of the results to human physiology (Bhatia and Ingber, 2014). Furthermore, OOC systems can be customized to mimic specific disease conditions, allowing researchers to study the mechanisms of disease and test potential therapeutics in a high-throughput manner (Esch et al., 2011).

These systems have been utilized in the studies of other infectious diseases, including malaria, tuberculosis, and influenza (Esch et al., 2014). These models recapitulate key aspects of host-pathogen interactions, such as the interaction between immune cells and pathogens, the dynamics of infection within host tissues, and the efficacy of antimicrobial drugs. For example, lung-on-chip systems have revealed important insights into pathogenesis of respiratory infections, including the recruitment of immune cells to the site of infection and the inflammatory response induced by the pathogen (Huh et al., 2011). Similarly, gut-on-a-chip models have uncovered the mechanisms of microbial colonization and host immune response to gut microbiota and enteric pathogens (Lu et al., 2018). Therefore, OOC systems represent a groundbreaking leap in biomedical research, offering unparalleled advantages by recreating human tissue environments with remarkable fidelity. This makes them particularly valuable in studying complex diseases such as schistosomiasis, where the lifecycle of the parasite and its effects on human tissues such as the liver and vasculature, are difficult to fully capture in existing models.

## Application of OOC systems in schistosomiasis research

In the context of schistosomiasis research, OOC technology offers a powerful platform for studying the mechanisms underlying the extravasation of schistosome eggs (Lu et al., 2018). By replicating the physiological conditions of the host vasculature and surrounding tissues, OOC models can provide insights into the interactions between schistosome eggs and the host immune system, as well as the factors that regulate egg extravasation (Costain et al., 2018). Furthermore, OOC platforms allow real-time imaging and analysis of cell responses, allowing for visualization and quantifying the dynamics of egg migration in a controlled experimental setting (Wu et al., 2020). This technology holds a great promise in advancing our understanding of schistosomiasis by providing a physiologically relevant platform to study the mechanisms underlying extravasation of *Schistosoma* eggs. Yeh et al., 2022, have demonstrated the utility of organ-on-chip models in elucidating the complex interactions between *Schistosoma* eggs and host tissues, as well as the factors that regulate egg extravasation (Yeh et al., 2022). By culturing endothelial cells in microfluidic channels and exposing them to schistosome eggs, we can simulate the initial stages of egg extravasation and investigate the mechanisms by which eggs breach the endothelial barrier. Past studies have revealed that schistosome eggs induce endothelial activation and permeability through the release of pro-inflammatory cytokines and chemokines, leading to the recruitment of immune cells to the site of infection (Angeles et al., 2020).

For example, a microfluidic system was developed by Girod et al., to mimic flow conditions in the human blood system that allowed the evaluation of drug effects on worm attachment and viability under dynamic conditions (Girod et al., 2022). The system demonstrated that healthy worms remained attached to the walls and resisted flow, while damaged worms were eliminated. This study highlights the importance of developing novel screening systems for the identification of schistosomicidal drugs contributing to the advancement of drug screening methodologies and provides a promising platform to evaluate the efficacy of other potential treatments against these parasites. Future advancements in this technology could implement automated worm counting for real-time analysis. OOC systems also enable real-time imaging and analysis of cellular responses, allowing researchers to visualize the dynamics of egg migration and granuloma formation in a controlled experimental setting. By incorporating advanced imaging techniques, such as confocal microscopy and live cell imaging, we can monitor the interactions between schistosome eggs and host cells in real time, providing insight into the spatiotemporal dynamics of egg extravasation (Singh et al., 2022). Furthermore, computational modeling approaches can be used to simulate the biophysical forces that regulate egg migration and predict optimal conditions for the formation of granulomas (King et al., 2021).

In addition to studying the mechanisms of egg extravasation, OOC models can be used to screen potential therapeutics targeting *Schistosoma* infection (Hong et al., 2024). By culturing immune cells and endothelial cells in microfluidic devices and exposing them to *Schistosoma* eggs, it is possible to evaluate the efficacy of anti-inflammatory drugs, immune modulators, and antiparasitic agents in preventing egg extravasation and granuloma formation (Mannino et al., 2018). These studies have identified several promising candidates for further preclinical testing, including small-molecule inhibitors of cytokine signaling pathways and monoclonal antibodies targeting immune cell receptors (Wynn and Cheever, 1995). Furthermore, OOC platforms can be used to study the long-term effects of *Schistosoma* infection on host tissues and organs (Colley et al., 2014). Culturing of cells from different organs, such as the liver, intestine, and bladder, in interconnected microfluidic devices, can model the systemic effects of chronic schistosomiasis and investigate the mechanisms underlying disease progression (Colley and Secor, 2014). These studies can uncover novel therapeutic targets to prevent or mitigate long-term complications of schistosomiasis, such as liver fibrosis and bladder cancer (Pearce and MacDonald, 2002).

## Using OOC systems to decipher the forces driving schistosome egg migration

Since schistosome eggs do not possess known motility mechanism, the puzzling and yet scientifically interesting question is how the eggs transit through layers of host tissues, including endothelial and epithelial barriers to reach the intestinal lumen and be excreted to continue the life cycle. As earlier stated, there is a better understanding of host immune interaction with the parasites, including characterization of proteins and enzymes such as matrix metalloproteinases (MMPs) and their tissue inhibitors (TIMPs) which are key regulators of extracellular matrix (ECM) turnover and remodeling in schistosomiasis infection (Page-McCaw et al., 2007). These play a critical role in both host defense and tissue pathology released by the parasites to aid in host invasion and immunomodulation for survival inside the host (Pearce and MacDonald, 2002).

The formation of granuloma is a complex process which involve infiltrations of alternatively activated macrophages, eosinophils, Th2 cells, fibroblast proliferation (Hams et al., 2013), angiogenesis, endothelial activation, and the release of blood clotting factors (Shariati et al., 2011; Mebius et al., 2013). As such, both *in vitro* and *in vivo* studies have reported that the timing and formation of granuloma are crucial to the successful translocation of schistosome eggs (Hams et al., 2013; Linder, 2017; Schwartz and Fallon, 2018; Takaki et al., 2021). These stromal cells play key roles in tissue repair, and become activated following tissue damage, as would happen during egg deposition (Gabbiani, 2003).

However, despite intense studies, precisely how the eggs transit through the complex granuloma ecosystem to reach the gut lumen remains largely unknown. Equally unclarified is the role of fibroblasts in granuloma formation and egg propulsion since fibroblasts are the most abundant cells in the stroma where eggs are laid. In normal development and physiology, fibroblasts are the major producers of the ECM. These stromal cells play key roles in tissue repair and become activated following tissue damage, as would happen during egg deposition (Gabbiani, 2003). During wound healing, these cells can produce transforming growth factor-β (TGFβ) and acquire a highly contractile phenotype associated with the expression of alpha smooth muscle antigen (αSMA) (Rockey et al., 2013). In this state, fibroblasts are termed ‘myofibroblasts’. Both in normal homeostasis and following injury they participate in crosstalk with adjacent epithelia, and their ability to influence local epithelial stem cell behavior has been documented (Brizzi et al., 2012; Le Guen et al., 2015). In addition, fibroblasts can also promote angiogenesis via the production of vascular endothelial growth factor A (VEGFA) and coordinate the function of the immune system via the production of chemokines and cytokines (Kraman et al., 2010; Buechler and Turley, 2018). Given the myriad roles of fibroblasts in homeostasis and being the most contractile among the cells within the granuloma microenvironment, it is likely that these cells provide thrust to the eggs. In support of this, earlier studies have pointed to the role of fibroblasts in granuloma formation in tuberculosis (TB) where they, confoundingly, provide both protection to the host as well as facilitate bacteria dissemination (Evans et al., 2019). Cancer associated fibroblasts (CAFs) are also suspected to produce contractile forces which drive cancer metastasis (reviewed in (Sahai et al., 2020)). Could a similar mechanism exist in schistosome egg dissemination, and if so, what is the exact role of fibroblasts in this process?

**Figure 3:**
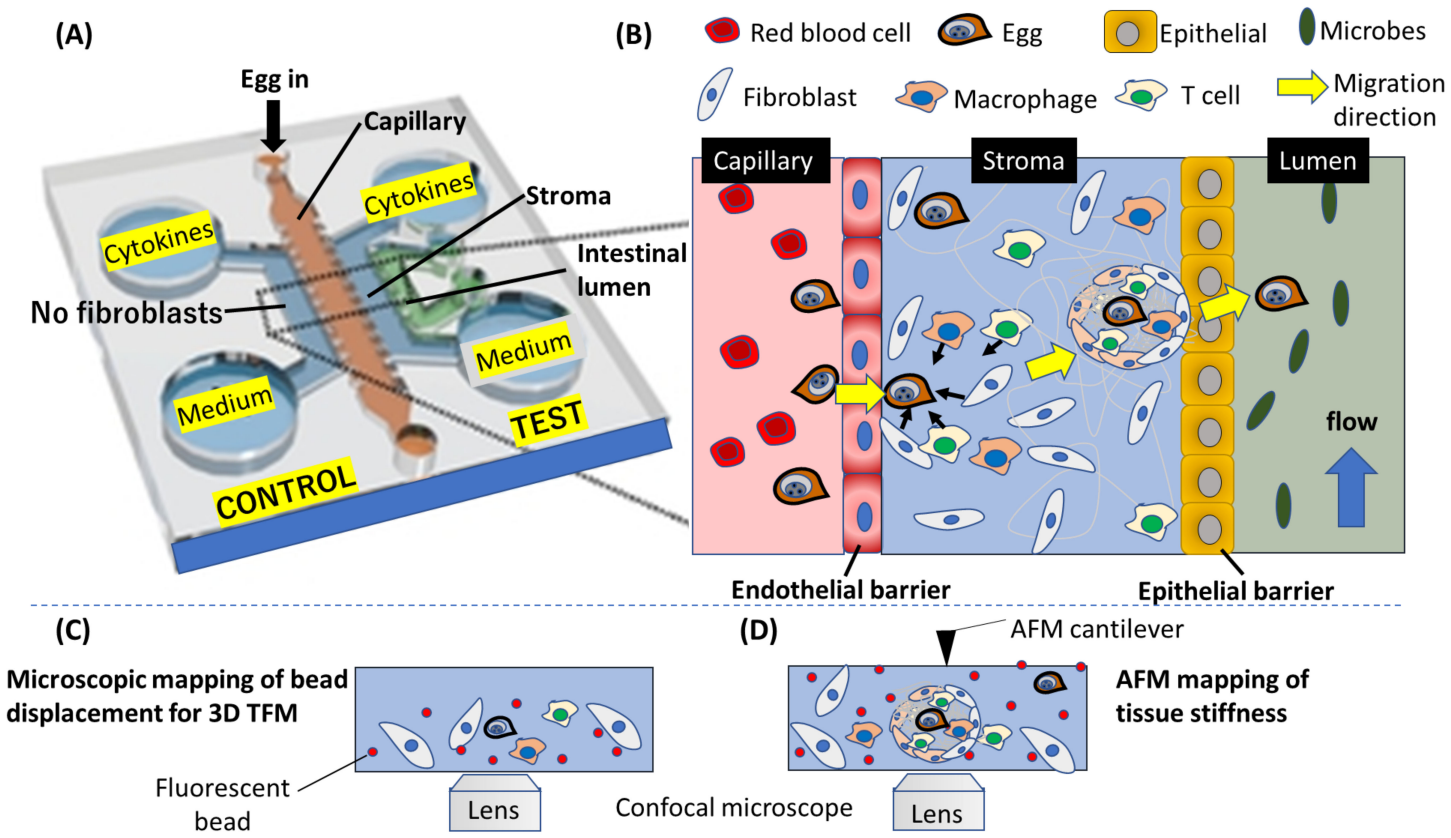
A conceptualized potential application of OOC to mimic schistosome egg migration through the gastrointestinal tissues. **(A)** A layout of the OOC showing the various components for tracking egg migration. **(B)** An enlarged diagram depicting the migration process on the OOC. The complex process involved in the egg migration, including the penetration of endothelial barrier wall, egg interaction with fibroblast and immune cells leading to the formation of granuloma in the stroma, and finally, egg transition through epithelial barrier into the intestinal lumen are depicted. **(C)** Fluorescence microscopy can be applied to track fluorescent beads embedded in the gel mimicking the stroma, and **(D)** force characterization using atomic force microscope (AFM) can yield quantitative information about the forces produced by host cells to propel Schistosome egg during the egg migration process. **(A)** is modified from Lee et al., 2019 (Lee et al., 2019).

OOC can be employed to clarify the migratory mechanism of schistosome eggs within gastrointestinal tissues from the perspective of tissue mechanics. Given the role of fibroblasts in remodeling the extracellular matrix (ECM) and generating contractile forces, we propose that egg-induced immune stimulation and/or ECM alterations—such as collagen and fibrin deposition and degradation—within the granuloma microenvironment may activate fibroblasts into a highly contractile myofibroblast phenotype. This activation could produce sufficient contractile forces to facilitate the migration of schistosome eggs. Thus, using *in vitro* studies utilizing microphysiological systems (MPS) as illustrated in **Figure 3**, which are now widely accepted as animal alternatives (Wikswo, 2014), we can take a tissue mechanistic approach to quantitatively map and dissect the nature and origin of forces that propel schistosome egg migration. Considering the roles of fibroblasts in matrix deposition and contractile force generation, we can use *in vitro* studies employing 3D MPS to determine the role of fibroblast-generated contractile forces in egg propulsion. Because cell contractility is closely associated with matrix stiffness such that on a stiffer matrix where cells can establish stronger focal adhesions, stronger contractile forces are generated compared with a soft matrix.

By adjusting the microenvironment in an OOC system, we can further investigate the conditions that promote or inhibit egg migration, distinguishing between “successful” (excreted) and “unsuccessful” (trapped) eggs. This allows for the identification of molecular and mechanical cues that influence egg migration outcomes. The OOC system can also mimic coagulation pathways by incorporating blood vessel-like structures that simulate vascular responses to egg transit. Introducing blood flow and clotting factors enables real-time observation of how coagulation and fibrinolysis are regulated during egg migration. Additionally, microfluidic channels can simulate blood vessels to explore clot formation, and how the fibrinolytic system clears clots to facilitate egg movement (Linder, 2017).

**Figure 4:**
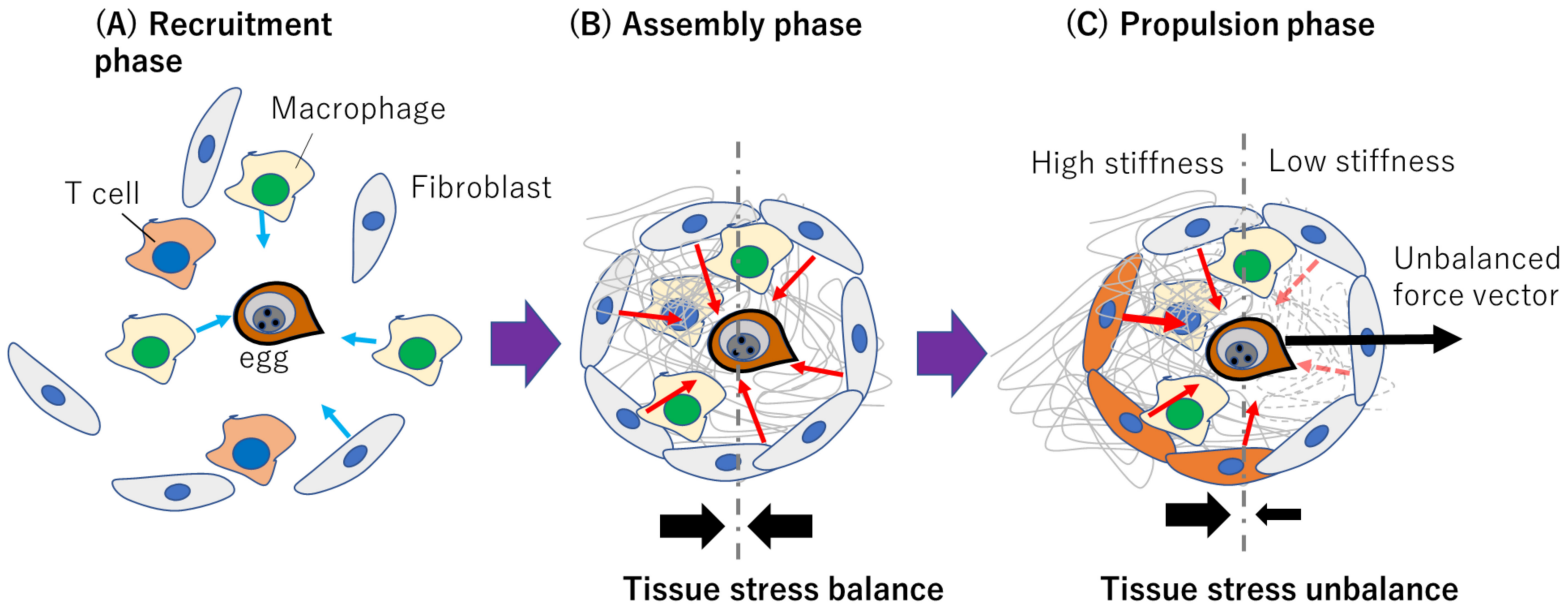
Conceptualized representation of how fibroblast-dependent tissue mechanics may drive schistosome egg migration. **(A)** Recruitment phase where immune cells and fibroblasts respond to egg released immunogenically. Light blue arrows indicate migration direction. **(B)** Assembly phase involving ECM deposition and isotropic contraction without a resultant force due to tissue stress balance. Red arrows indicate hypothesized fibroblast-generated contractile forces within the granuloma **(C)** Propulsion phase where polarized ECM degradation or myofibroblast differentiation contributes to anisotropic contraction and egg propulsion. The red dashed arrows indicate diminishing forces due to matrix degradation. The solid black arrows in **(B, C)** represent the tissue stress balance or unbalance.

**Figure 5:**
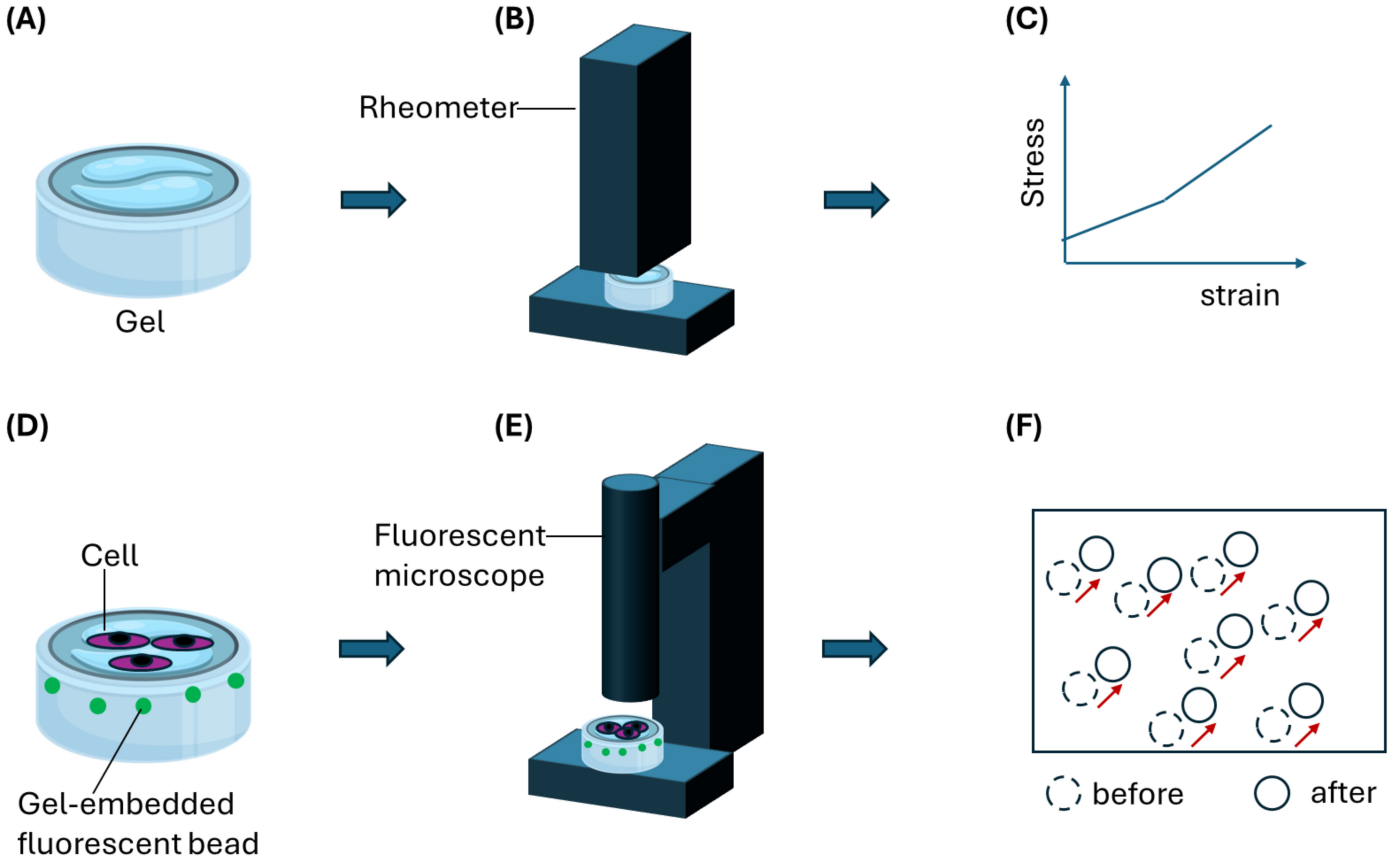
An illustration of a gel-based assay to decipher forces produced by host cells such as contractile fibroblasts to aid Schistosoma eggs migration. **(A)** A hydrogel is first characterized using **(B)** stiffness measurement equipment such as AFM or rheometer. **(C)** A stress-strain relationship obtained from the characterization is then used to derive quantitative stiffness information such as Young’s modulus. **(D)** Gel samples can be embedded with fluorescence particles such as beads for optical tracking and mapping of gel deformation during cell migration on or through the gel. Schistosoma eggs can be co-cultured on the gel surface in contact with host cells such as fibroblasts. **(E)** Tracking bead displacement using a fluorescent microscope would yield **(F)** a displacement field resulting from gel deformation. By applying particle image velocimetry (PIV) analysis, migration forces produced by host cells can be determined quantitatively.

Thus, we hypothesize that when egg-induced matrix degradation occurs due to egg-secreted matrix metalloproteinase (MMP) (Singh et al., 2004; Singh et al., 2006), fibroblasts on the degraded part of the granuloma can exert less contractility compared with those on the unaffected part, creating an anisotropic contraction which may push the eggs out of the granuloma towards the blood vessels as shown in **Figure 4**. To clarify this, in 3D MPS, we can deploy high resolution live cell tracking, 3D force mapping techniques, and tissue mechanics, coupled with immunological characterization and genetic profiling to characterize fibroblast-egg interaction right from the initiation of granuloma formation to the egg migration stage. 3D MPS can recapitulate the gastrointestinal microenvironment where we could deploy it to spatiotemporally and quantitatively study the role of fibroblasts in the formation of granuloma and, eventually, egg migration. Leveraging on tissue mechanics, we can map forces within the granuloma microenvironment, using quantitative approaches integrating 3D traction force microscopy (3D TFM) and atomic force microscopy (AFM) measurements. From cellular migration and tissue deformation analyses, we could generate a force map to reveal the force vector dictating the trajectory of egg migration within the gastrointestinal ecosystem as depicted in **Figure 5**. 3D MPS can therefore be used to map the nature and origin of the forces that propel egg migration, with focus on fibroblasts.

## Conclusion

Over the past few decades, research has revealed many fascinating aspects of *S. mansoni* biology and the host’s immune response to both the worms and their eggs. The immunology of granuloma formation around the eggs has been a major focus, given its central role in the immunopathology of schistosomiasis infections. Despite significant advances, many unknown aspects remain to be identified and experimentally validated. One such area is the process by which granulomas evolve to facilitate egg migration through the intestinal wall, liver, spleen and other organs. Thus, a 3D MPS can recapitulate the gastrointestinal microenvironment where we could deploy it to spatiotemporally and quantitatively study the role of immune cells and fibroblasts in the formation of granuloma and, eventually, egg migration. Leveraging on tissue mechanics, we can map forces within the granuloma microenvironment, using quantitative approaches integrating 3D TFM and AFM measurements. From cellular migration and tissue deformation analyses, we could generate a force map to reveal the force vector dictating the trajectory of egg migration within the gastrointestinal ecosystem. 3D MPS can therefore be used to map the nature and origin of the forces that propel egg migration, with focus on immune cells and fibroblasts. This cutting-edge platform, which mimics the structure and function of human organs, could allow for detailed investigation of egg transit in a controlled environment.
